# The Collection of Adipose Derived Stem Cells using Water–Jet Assisted Lipoplasty for their Use in Plastic and Reconstructive Surgery: A Preliminary Study

**DOI:** 10.3389/fcell.2016.00136

**Published:** 2016-11-22

**Authors:** Valeria Purpura, Elena Bondioli, Davide Melandri, Pier C. Parodi, Luca Valenti, Michele Riccio

**Affiliations:** ^1^Burn Center and Emilia Romagna Regional Skin Bank, “M.Bufalini” Hospital, AUSL della RomagnaCesena, Italy; ^2^Clinic of Plastic and Reconstructive Surgery, University of Udine, “AOU Santa Maria della Misericordia”Udine, Italy; ^3^Clinic of Plastic and Reconstructive Surgery, University of Milano, “IRCSS San Donato”Milano, Italy; ^4^S.O.D.C. Plastic and Reconstructive Hand Surgery, “Ospedali Riuniti” HospitalAncona, Italy

**Keywords:** fat graft, adipose stem cells, regenerative surgery, water–jet lipoplasty, stromal vascular fraction

## Abstract

The graft of autologous fat for the augmentation of soft tissue is a common practice frequently used in the field of plastic and reconstructive surgery. In addition, the presence of adipose derived stem cells (ASCs) in adipose tissue stimulates the regeneration of tissue in which it is applied after the autologous fat grafting improving the final clinical results. Due to these characteristics, there is an increasing interest in the use of ASCs for the treatment of several clinical conditions. As a consequence, the use of clean room environment is required for the production of cell-based therapies. The present study is aimed to describe the biological properties of adipose tissue and cells derived from it cultured *in vitro* in clean room environment according to current regulation. The collection of adipose tissue was performed using the water–jet assisted liposuction in order to preserve an high cell viability increasing their chances of future use for different clinical application in the field of plastic and reconstructive surgery.

## Introduction

Nowadays, the use of therapies based on the application of adult stem cells is a growing perspective for the treatment of different clinical conditions (Trounson and Mc Donald, 2015). In fact, stem cell based therapies have the potential to give relevant scientific results improving clinical conditions of patients affected by different diseases. For this reason, several funds were addressed to the development of this research field with the production of different clinical trials (Trounson and Mc Donald, 2015). On the other hand, it is a relatively new research field and final outcomes are not still available for their translation into clinical practice. As a consequence, the transplant of bone marrow is actually the only procedure in which adult stem cells derived from blood are routinely used for clinical applications since the 1960s, after evidences of the beneficial effects of cells derived from bone marrow on irradiated mice (Jacobson et al., 1951). It can be explained by the ability of stem cells to home in a damaged tissue of the body in which they can induce regeneration through their differentiation in tissue specific cell type as well as by the secretion of different growth factors involved in growth of resident cells. In particular, the mesenchymal stem cells (MSCs) are considered as a main available reservoir of multipotent progenitor cells able to differentiate *in vitro* in several tissues such as cartilage or bone as well as fat or muscle (Hauner et al., 1987; Grigoradis et al., 1988; Johnson et al., 1988; Wakitani et al., 1995; Ferrari et al., 1998; Pittenger et al., 1999) and to be isolated by different tissues. For these characteristics, MSCs were taken into account for the treatment of different clinical conditions and several clinical trials for their use in stem cell therapy are actually in progress (Wang et al., 2012). On the other hand, the collection of MSCs from some tissues such as adipose tissue results less invasiveness compare to their collection from bone marrow. In fact, adipose derived stem cells (ASCs) can be retrieved easily using liposuction aspirates and they can be easily expanded and differentiate *in vitro* toward different lineages (Zuk et al., 2002). The collection of adipose tissue using liposuction aspirates is a simple, reproducible and low-risk method frequently used in the field of plastic and reconstructive surgery (Pu, 2012). In particular, the water–jet assisted liposuction (Ueberreiter et al., 2010; Hoppe et al., 2013) is not only a new technique for the collection of adipose tissue but a whole new concept of liposuction. In fact, it is able to preserve cell viability in addition to be a painless and bloodless procedure able to produce less tissue trauma compared to traditional liposuction aspirates (Araco et al., 2007; Yin et al., 2015). Thus, due to their characteristics and easy collection, ASCs can be widely used for the treatment of a broad range of clinical conditions. Considering the increasing interest in the development of cell therapies using ASCs, tissue processing in clean room environment is required.

Here, we described the biological properties of adipose tissue and cells derived from it cultured *in vitro* in clean room environment according to current regulation. The collection of adipose tissue was performed using the water–jet assisted liposuction in order to preserve an high cell viability enhancing their chances of future use for different clinical application in the field of plastic and reconstructive surgery.

## Materials and methods

### Adipose tissue procurement

**Figure 1:**
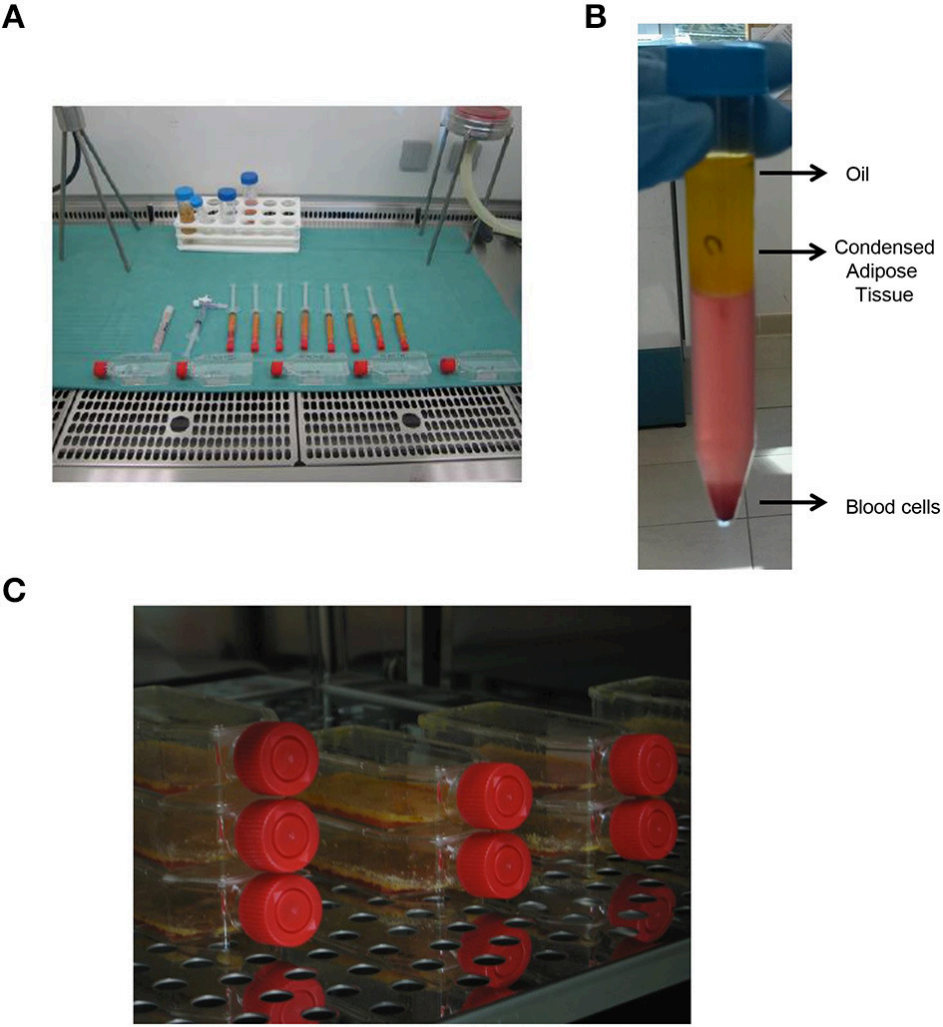
**Procurement and culture *in vitro* of human adipose tissue. (A)** Procurement of human adipose tissue and its processing under laminar flow hood in class B GMP. **(B)** Centrifugation of human adipose tissue in order to obtain condensed adipose tissue. **(C)** *In vitro* culture of human adipose tissue at 37°C.

Human adipose tissue has been received from the Department of Reconstructive and Plastic Hand Surgery of “Ospedali Riuniti” of Ancona (Figure 1A), and all experimental procedures were then carried out at Emilia Romagna Regional Skin Bank, AUSL, Romagna. In all our cases, human adipose tissue has been aspirated by using the water–jet assisted liposuction. Adipose tissue was then washed in sterile saline solution (0.9% NaCl, Fresenius Kabi) and centrifuged at 2000 rpm for 5 min at 25°C in order to discard blood cells as well as oil (Figure 1B). The condensed adipose tissue was then cultured in RPMI 1640 medium with penicillin (10,000 IU/ml), streptomycin (10 mg/ml), amphotericin B (25 μg/ml), and 10% of fetal bovine serum (FBS) at 37°C in an atmosphere of 5% CO_2_/air (Figure 1C).

### Cell viability test (MTT)

Human adipose tissue used for *in vitro* culture was weighed and placed in a multi-well plate. Then, it was incubated with a solution of MTT 0.5 mg/ml (Roche Diagnostic GmbH, Penzberg, Germany) for 3 h at 37°C in an atmosphere of 5% CO_2_/air. Then, each sample was placed in 1 ml of dimethyl sulphoxide (DMSO) for 10 min and the resulting colored solution was read at 570 nm using a spectrophotometer. The level of absorbance of DMSO was used as blank. The viability rate of each sample was then evaluated as the ratio between the optical density (OD) at 570 nm and the weight in grams of tissue (g).

### Microbiological analysis

For microbiological analysis, adipose tissue was plated immediately (Adipose tissue To) or after 14 days of *in vitro* culture (Adipose tissue 14 days) on plates selective for growth of bacteria (COS Columbia agar + 5% sheep blood, BioMerieux) or fungi (Sabouraud dextrose agar + CAF, Biolife). Microbiological analysis were then performed on plates after their incubation at 32.5°C for, respectively, 3 or 14 days.

### *In vitro* culture of ASC

Human adipose tissue was digested with collagenase 0.075% for 1 h at 37°C in order to isolate stromal vascular fraction (SVF) containing ASCs. Adipose tissue has been also shaked every 10 min in order to improve the efficiency of digestion. Then, the digested adipose tissue was centrifugated at 2000 rpm for 5 min at 25°C and the pellet containing ASCs in SVF was washed with saline solution and cultured in RPMI 1640 medium with penicillin (10,000 IU/ml), streptomycin (10 mg/ml), amphotericin B (25 μg/ml), and 10% of FBS.

## Results

**Figure 2:**
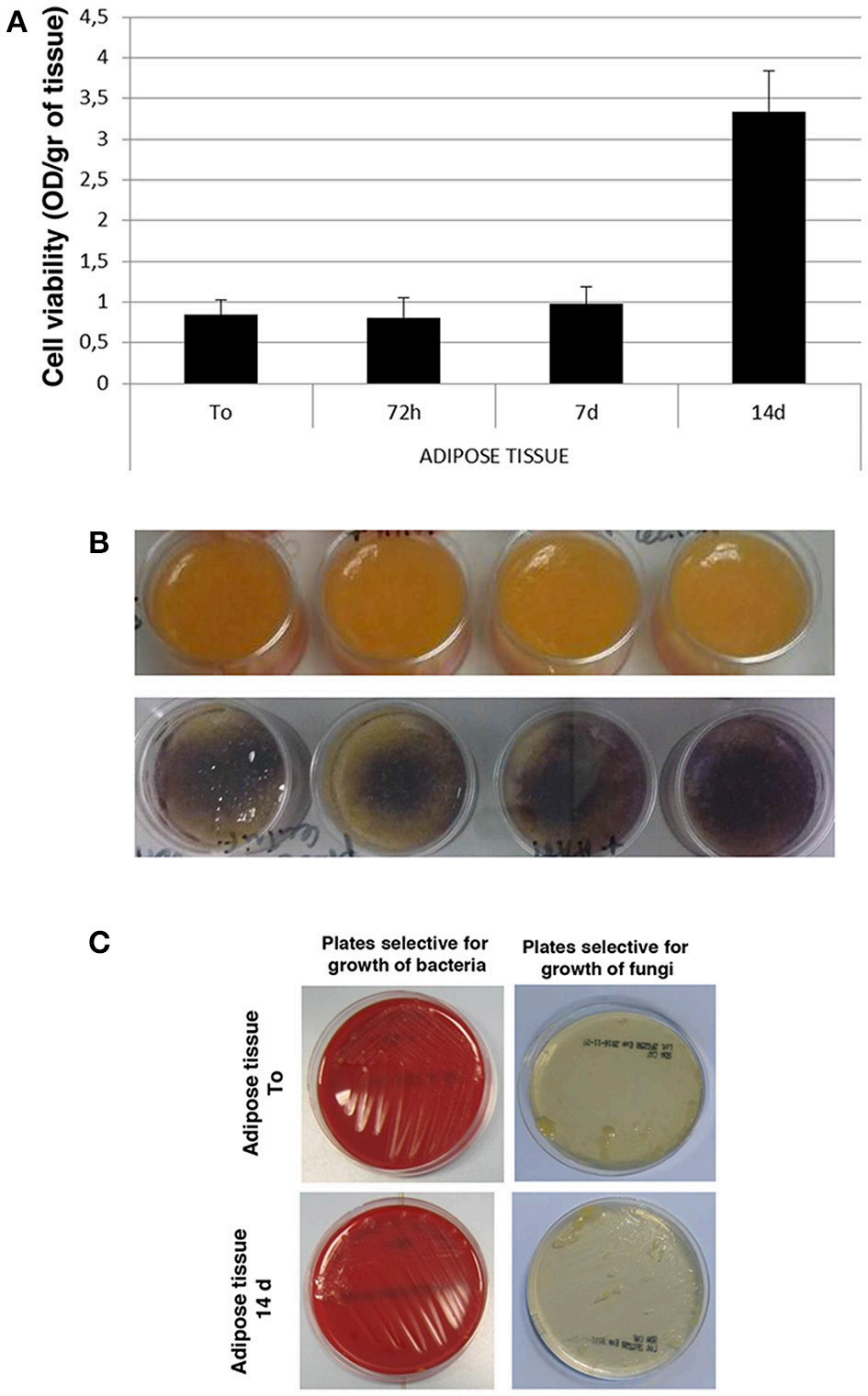
**Cell viability and microbiological analysis of human adipose tissue. (A)** Viability rate of human adipose tissue at different times of *in vitro* culture. **(B)** Morphological analysis of human adipose tissue before (upper panel) and after (bottom panel) incubation with MTT solution. **(C)** Microbiological analysis on human adipose tissue immediately (Adipose tissue To) and after 14 days (Adipose tissue 14 days) of culture on selective plates.

Starting from human adipose tissue aspirated by using the water–jet assisted liposuction, all the experimental procedures were carried out in clean room environment of cell factory according to current regulation. Thus, all samples of adipose tissue were processed in sterile conditions under laminar flow hood in class B GMP (Figure 1A). Samples of no-digested adipose tissue were washed with saline solution and centrifugated in order to discard blood cells as well as oil (Figure 1B), and finally they were cultured *in vitro* at high density for different times (Figure 1C), in order to perform different analysis. In particular, we performed an *in vitro* evaluation of cell viability in adipose tissue immediately (To) and after 72 h as well as 7 and 14 days of culture using the MTT-test. As shown in Figure 2A, the viability of cells in adipose tissue is maintained similar up to 7 days and it increases significantly after 14 days demonstrating that cells in adipose tissue are able to proliferate in *in vitro* culture conditions. In addition, viable cells contained in adipose tissue provide to it a purple color after the incubation with MTT for the production of formazan in metabolically active cells (Figure 2B, bottom panel) differently from no-treated adipose tissue (Figure 2B, upper panel), as expected. In order to evaluate if sterile conditions have been maintained after different times of cell culture, a small amount of adipose tissue was placed on plates selective for growth of bacteria or fungi both immediately (Adipose tissue To) and after 14 days (Adipose tissue 14 days) of culture using gamma sterilized inoculating loops. After an incubation of 3 and 14 days at 32.5°C, we identified that sterile conditions were maintained since no growth of bacterial and fungi was evident in both plates used (Figure 2C).

**Figure 3:**
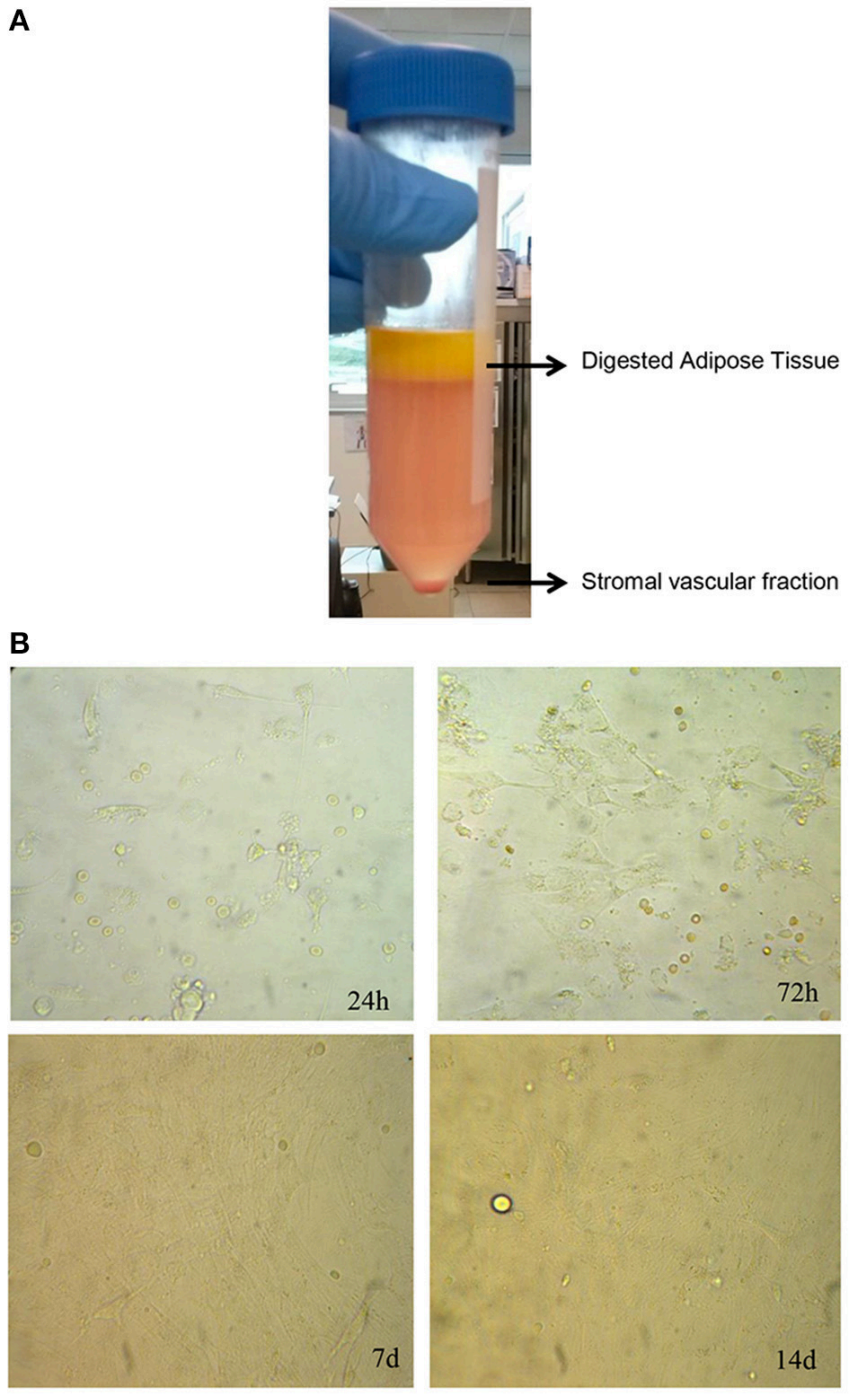
**Isolation and culture of stromal vascular fraction. (A)** Enzymatic digestion of human adipose tissue to isolate stromal vascular fraction. **(B)** Morphological analysis at different times of stromal vascular fraction cultured *in vitro*.

**Figure 4:**
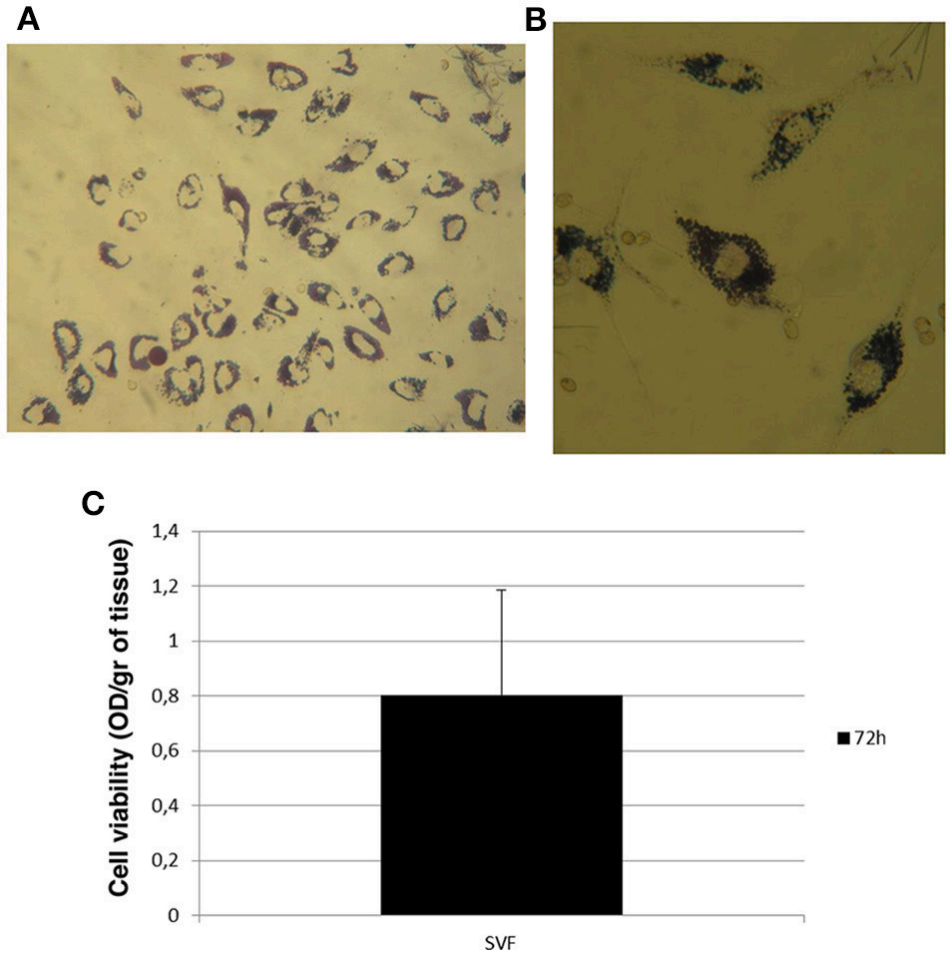
**Cell viability of stromal vascular fraction at 72 h. (A)** Morphological evaluation of cells in stromal vascular fraction after incubation with MTT solution. **(B)** Magnification of cells containing formazan showed in panel **(A)**. **(C)** Viability rate of stromal vascular fraction after 72 h of *in vitro* culture.

Since adipose tissue showed viable cells during *in vitro* culture, we decided to evaluate if their viability could be maintained after enzymatic digestion of adipose tissue with the collagenase in order to culture and enhance cells *in vitro*. To this aim, we digested adipose tissue following the protocol described in Section Materials and Methods in order to obtain the SVF, also containing the ASCs (Figure 3A). After its procurement, SVF was cultured *in vitro* and a morphological analysis was performed at different times. As shown in Figure 3B, cells contained in SVF show a different morphology over the experimental time with a roundish appearance after 24 h that resulted stretched after 72 h probably as a consequence of cell adhesion to the culture dish underlying their ability to growth in culture. After 7 and 14 days, cells reaching the confluence and appeared as large cells difficult to be identified at optical microscope (Figure 3B). Then, we evaluated the viability of SVF cultured *in vitro* for 72 h since it appeared an experimental time in which cells adhered well to culture flask without reaching the confluence. Thus, adipose tissue was enzymatically digested to obtain SVF. Then, SVF was cultured *in vitro* for 72 h after which cells were incubated with MTT solution for 3 h at 37°C. As shown in Figures 4A,B, viable cells treated with MTT solution showed the formation of formazan with purple color after 3 h of incubation, demonstrating their viability after 72 h of *in vitro* culture. In fact, MTT-test is a colorimetric assay that assess cell viability taking advantage of the NAD(P)H-dependent cellular oxidoreductase enzyme ability to reduce the yellow tetrazole (MTT) to insoluble purple formazan in living cells. Thus, the formazan formation is directly proportional to cell viability. The formazan produced inside cells is also more evident in the magnification showed in the Figure 4B. To quantify this result we then dissolved insoluble purple formazan with DMSO and we quantify the values of absorbance of the colored solution obtained by a spectrophotometer. The graph in Figure 4C shows values of absorbance comparable with that identified in no-digested adipose tissue demonstrating, in turn, that an equal amount of viable cells could be obtained in *in vitro* culture after digestion of adipose tissue.

## Discussion

The study here described is aimed to evaluate the biological characteristics of adipose tissue and cells derived from it in *in vitro* culture condition in order to evaluate their potential future use as cell-based therapy for different clinical applications in the field of plastic and reconstructive surgery. In order to obtain appreciable results, we aspirated the adipose tissue by using the water–jet assisted liposuction, a bloodless technique able to preserve cell viability during liposuction (Araco et al., 2007; Ueberreiter et al., 2010; Yin et al., 2015). In fact, the collection of adipose tissue using no traumatic technique is preferred to traditional liposuction as previously described (Pu, 2012). Several authors previously evaluated the effectiveness of different methods used for the collection of adipose tissue taking into account cell viability in it immediately after its procurement. On the other hand, the use of different assays and protocols for the processing of adipose tissue in order to analyze cell viability makes results not comparable (Pu, 2012). In our study, we focused our attention on viability of both no-digested adipose tissue and cells derived from it after its enzymatic digestion during their culture *in vitro* at different experimental times since future application in cell based therapy was of interest for us. To this aim, we decided to condense adipose tissue after its procurement using centrifugation in order to increase their chances of survival during the culture *in vitro*. In fact, it was previously described as a procedure able to increase the amount of viable cells (Boschert et al., 2002) as well as growth factors (Pallua et al., 2009) in addition to clean adipose tissue from blood cells as well as oil. On the other hand, we chose an intermediate force for centrifugation of adipose tissue since too high centrifugal force is not recommended for the maintenance of cell viability in it (Xie et al., 2010). Our results showed high levels of cell viability in adipose tissue immediately after its collection that significantly increased after 14 days of *in vitro* culture. Then, we also performed our analysis on cells derived from adipose tissue after its digestion. Thus, the digestion of adipose tissue allowed us to isolate viable and proliferating cells in *in vitro* culture. Also in this case, we selected a centrifugal force previously used in order to avoid damage of cells (Bunnell et al., 2008). Similar to previous morphological studies on SVF, our results showed adherent cells on dish after 72 h of culture with a spindle shape that reached the confluence after 14 days of *in vitro* culture (Choudhery et al., 2014). Thus, cells derived from digestion of adipose tissue corresponding to SVF maintain a viability comparable to no-digested adipose tissue and can be cultured and enhanced in *in vitro* culture. Considering the potent regenerative capacity of ASCs contained in the SVF and their ability to improve the healing process in damaged tissue after autologous fat grafting, the maintenance of cell viability after enzymatic digestion of adipose tissue as well as their culture and enhancement *in vitro* can be considered as an useful tool for their potential future clinical use in the field of plastic and reconstructive surgery. On the other hand, more experimental analysis to study in depth their characteristics in *in vitro* culture conditions should be performed also taking into account the abnormalities previously identified after several passages *in vitro* by other authors (Rubio et al., 2005).

Thus, we can conclude that our preliminary data support further studies on SVF for its ability to be maintained viable in *in vitro* culture conditions after digestion of human adipose tissue according to our protocol. Thus, the culture and enhancement in clean room environment of cellular component derived from adipose tissue could be used as a future cell therapy in order to improve fat grafting and, in turn, clinical results in the field of plastic and reconstructive surgery.

## Author contributions

Substantial contributions to the conception or design of the work; or the acquisition, analysis, or interpretation of data for the work: VP, EB, DM, MC. Drafting the work or revising it critically for important intellectual content: PP, LV. Final approval of the version to be published: MR.

### Conflict of interest statement

The authors declare that the research was conducted in the absence of any commercial or financial relationships that could be construed as a potential conflict of interest. The reviewer GF and handling Editor declared their shared affiliation, and the handling Editor states that the process nevertheless met the standards of a fair and objective review.
